# Systematic evaluation of cancer risk associated with rs2292832 in miR-149 and rs895819 in miR-27a: a comprehensive and updated meta-analysis

**DOI:** 10.18632/oncotarget.8082

**Published:** 2016-03-14

**Authors:** Yajing Feng, Fujiao Duan, Chunhua Song, Xia Zhao, Liping Dai, Shuli Cui

**Affiliations:** ^1^ Department of Infection Management, The First Affiliated Hospital of Zhengzhou University, Zhengzhou, 450052, Henan, P.R.China; ^2^ Department of Hospital Infection Management, Affiliated Cancer Hospital of Zhengzhou University, Henan Cancer Hospital, Zhengzhou 450008, Henan, P.R.China; ^3^ Department of Epidemiology, College of Public Health, Zhengzhou University, Zhengzhou 450001, Henan, P.R.China; ^4^ College of Professional Study, Northeastern University, Boston, 02215 Massachusetts, USA

**Keywords:** miR-149, miR-27a, cancer, susceptibility, systematic evaluation

## Abstract

The aim of this study is to provide a precise quantification for the association between miR-149 T > C (rs2292832) and miR-27a A > G (rs895819) and the risk of cancer. We conducted a systematic literature review and evaluated the quality of included studies based on Newcastle-Ottawa Scale (NOS). Pooled odds ratios (ORs) and corresponding 95% confidence intervals (95% CIs) were calculated to assess the strengths of the associations. We identified 40 studies for pooled analyses. Overall, the results demonstrated that the rs2292832 polymorphism was subtly decrease the risk of breast cancer (CT + CC vs TT: OR = 0.83, 95% CI: 0.70–0.98, *P* = 0.03; CC vs CT + TT: OR = 0.80, 95% CI: 0.68–0.93, *P* = 0.00), and the rs895819 polymorphism wasassociated with significantly increased cancer risk in the Asian population (AG + GG vs AA: OR = 1.24, 95% CI: 1.03–1.50, *P* = 0.02) and in colorectal cancer subgroup (GG vs AA: OR = 1.45, 95% CI: 1.10–1.92, *P* = 0.00; AG + GG vs AA: OR = 1.35, 95% CI: 1.15–1.58, *P* = 0.00; GG vs AG + AA: OR = 1.36, 95% CI: 1.04–1.77, *P* = 0.02). In addition, a subtly decreased risk was observed in the Caucasian population and in breast cancer subgroup. In conclusion, the rs2292832 polymorphism was significantly associated with increased breast cancer risk, and the rs895819 polymorphism contributes to the susceptibility of colorectal and breast cancer.

## INTRODUCTION

MicroRNAs (miRNAs) are a group of short noncoding RNAs of about 22 nucleotides which are involved in diverse physiological and developmental processes by controlling the gene expression of target mRNAs [1, 2]. Accumulating evidence has shown that miRNAs regulate the expression of roughly 10–30% of the all human genes through post-transcriptional mechanisms [3], contributing to excessive physiologic and pathologic conditions, including cell differentiation, apoptosis, development, and deregulation of these processes play critical roles in carcinogenesis [4].

Single nucleotide polymorphisms (SNPs) represent the most common genetic variation in human genome. SNPs in miRNA genes are regarded to affect function by three ways: first, through the transcription of the primary transcript; second, through pri-miRNA and pre-miRNA processing; and third, through effects on miRNA-miRNA interactions [5]. Recently, several studies have demonstrated that some SNPs present in the miRNA genes [6, 7], which can alter miRNA expression and/or maturation and be associated with the development and progression of cancer [8]. Thus, SNPs in miRNAs may influence susceptibility to malignant tumors. The miR-149 T > C (rs2292832) and miR-27a A > G (rs895819) were studied in diverse cancers. Research results about two sites were inconsistent [9, 10], this discrepancy maybe partially attributed to the heterogeneity of the cancer subtype, small sample size, and ethnicity of the patients.

To further determine whether there is an association of the rs2292832 and rs895819 in the miRNA genes with the risk for developing cancer, a comprehensive review and analysis of published data from different studies is needed. In this study, we performed a meta-analysis on all eligible case-control studies to drive a more powerful estimation of the association of rs2292832 and rs895819 SNP with cancer risks.

## RESULTS

### Study characteristics

**Figure 1:**
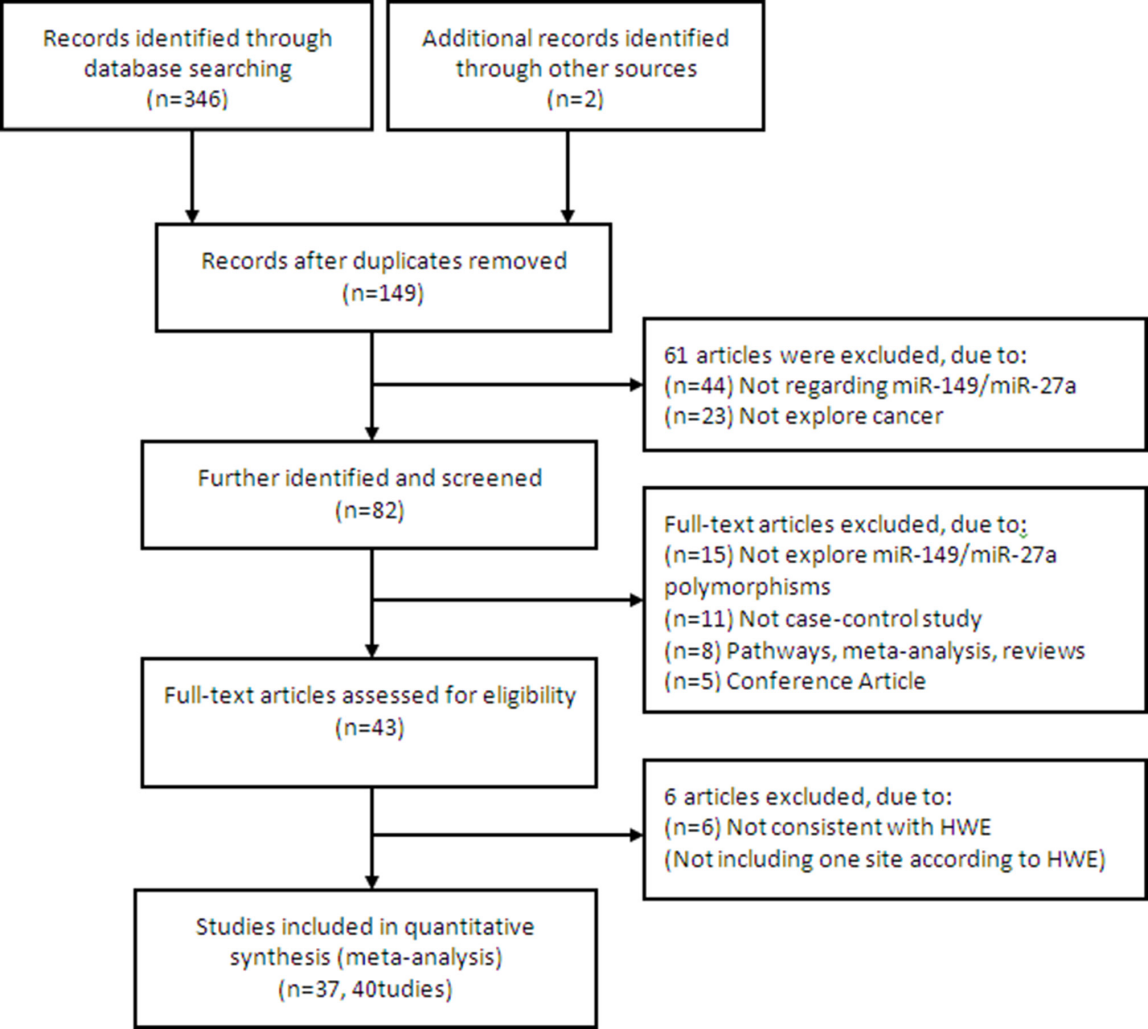
Flow chart of literature search and study selection

The search process and the final selection of relevant studies are shown in Figure 1, A comprehensive literature search yielded 348 potentially relevant published articles. After further identification and screening individual study, 43 articles (49 studies) [11–53] underwent full-text assessment, and 6 articles (10 studies, not including one site according to HWE) [14, 17, 19, 20, 35, 42] were excluded due to inconsistently with HWE. Finally, 37 articles (40 studies) [11–13, 15, 16, 18, 21–34, 36–41, 43–53] were conducted in quantitative synthesis.

Characteristics of included studies are presented in Table 1. A total of 39 eligible studies met the prespecified inclusion criteria, in which two articles [24, 52] included two tumor types respectively, and one article included [23] rs2292832 and rs895819. As for rs2292832, involving 9,994 cases and 10,757 controls were ultimately analyzed from 21 studies (20 articles) [11–13, 15, 16, 18, 21–34], and 19 studies (17 articles) [23, 36–41, 43–53] involving 7,800 cases and 9,060 controls for rs895819.

**Table 1:** Main characteristics of included studies

First author	Year	Ethnicity	Cancer type	Source of control	Genotyping	Match^a^	Sample size	*P*_HWE_		Quality control
						Y/N	Case/Control	rs2292832	rs895819	
He BS [11]	2015	Asian	Breast cancer	Population	MassARRAY	Y	450/450	0.13		Y
Du ML [12]	2014	Asian	Renal cell cancer	Population	TaqMan	Y	355/362	0.46		Y
Dikeakos P [13]	2014	Caucasian	Gastric cancer	Hospital	PCR-RFLP	Y	163/480	0.45		Y
Pu JY [14]	2014	Asian	Gastric cancer	Hospital	PCR-RFLP	N	220/530	< 0.01		Y
Wei WJ [15]	2014	Asian	PTC	Population	MassARRAY	Y	838/1006	0.73		Y
Wang R [16]	2014	Asian	HCC	Population	MassARRAY	N	944/984	0.86		N
Wu RR [17]	2014	Asian	Colorectal Cancer	Hospital	ASA	N	175/300	< 0.01	0.02	Y
Huang GL [18]	2013	Asian	NPC	Population	PCR-RFLP	N	158/242	0.72		Y
Chu YH [19]	2013	Asian	HCC	Population	PCR-RFLP	N	188/337	< 0.01		Y
Lv M [20]	2013	Asian	Colorectal cancer	Population	PCR-RFLP	N	353/540	< 0.01		Y
Song XC [21]	2013	Caucasian	OSCC	Population	PCR-RFLP	Y	325/335	0.99		Y
Tu HF [22]	2012	Asian	HNSCC	Hospital	PCR-RFLP	N	122/273	0.27		NA
Zhang M [23]	2012	Asian	Breast Cancer	Population	PCR-RFLP	Y	252/248	0.21	0.12	Y
Zhang MW(C) [24]	2012	Asian	Colorectal Cancer	Population	PCR-RFLP	Y	443/435	0.43		Y
Zhang MW(G) [24]	2012	Asian	Gastric Cancer	Population	PCR-RFLP	Y	274/269	0.70		Y
Min KT [25]	2012	Asian	Colorectal Cancer	Population	PCR-RFLP	N	446/502	0.62		Y
Ahn DH [26]	2012	Asian	Gastric Cancer	Population	PCR-RFLP	N	461/447	0.98		Y
Kim WH [27]	2012	Asian	HCC	Population	PCR-RFLP	N	159/201	0.34		Y
Vinci S [28]	2013	Caucasian	Colorectal Cancer	Population	HRM	Y	160/178	0.91		Y
Vinci S [29]	2011	Caucasian	Lung Cancer	Population	HRM	Y	101/129	0.97		Y
Li PY [30]	2011	Asian	NPC	Hospital	TaqMan	Y	791/1016	0.49		NA
Zhang MW [31]	2011	Asian	Lung Cancer	Population	PCR-RFLP	Y	232/231	0.12		Y
Liu ZS [32]	2010	Caucasian	HNSSC	Population	PCR-RFLP	Y	1109/1130	0.72		Y
Tian T [33]	2009	Asian	Lung Cancer	Population	PCR-RFLP	Y	1058/1035	0.86		Y
Wang ZW [34]	2009	Asian	Breast Cancer	Population	PCR-RFLP	Y	1009/1093	0.16		Y
Ma JY [35]	2015	Asian	NSCC	Population	TaqMan	Y	542/557		0.02	Y
Qi P [36]	2015	Asian	Breast cancer	Population	TaqMan	Y	321/290		0.69	N
Yin ZH [37]	2015	Asian	Lung Cancer	Hospital	TaqMan	Y	258/310		0.70	Y
Cao Y [38]	2014	Asian	Colorectal cancer	Population	PCR-RFLP	Y	254/238		0.09	Y
Kupcinskas J (C) [39]	2014	Caucasian	Colorectal cancer	Hospital	TaqMan	N	193/428		0.24	Y
Kupcinskas J (G) [40]	2014	Caucasian	Gastric cancer	Hospital	TaqMan	N	363/351		0.15	Y
Song B [41]	2014	Asian	Gastric cancer	Population	TaqMan	Y	278/278		0.11	Y
Wang ZQ [42]	2014	Asian	Colorectal cancer	Hospital	TaqMan	N	205/455		< 0.01	Y
Zhang JJ [43]	2014	Asian	ESCC	Population	SNaPshot	Y	1109/1275		0.23	Y
Zhang N [44]	2013	Asian	Breast cancer	Population	TaqMan	Y	264/255		0.45	N
Catucci I [45]	2012	Caucasian	Breast Cancer	Hospital	TaqMan	Y	1,025/1,593		0.051	Y
Hezova R [46]	2012	Caucasian	Colorectal Cancer	Population	TaqMan	Y	197/202		0.87	NA
Shi DN [47]	2012	Asian	Renal Cell Cance	Population	TaqMan	Y	594/600		0.37	Y
Zhang MW [48]	2012	Asian	Colorectal Cancer	Population	PCR-RFLP	Y	463/468		0.35	Y
Zhou Y [49]	2012	Asian	Gastric cancer	Hospital	MassARRAY	Y	311/425		0.94	Y
Zhang P [50]	2011	Asian	Breast Cancer	Population	MassARRAY	Y	384/192	< 0.01	0.61	Y
Sun QM [51]	2010	Asian	Gastric cancer	Hospital	PCR-RFLP	Y	304/304		0.053	Y
Kontorovich T(B) [57]	2010	Caucasian	Breast cancer	Population	iPLEX	N	86/106	< 0.01	0.37	Y
Kontorovich T(O) [52]	2010	Caucasian	Ovarian cancer	Population	iPLEX	N	34/106	< 0.01	0.37	Y
Yang RX [53]	2010	Caucasian	Breast cancer	Population	TaqMan	Y	1189/1416		0.14	Y

a Match, controls and cases were matched on age and gender; ASA, allele-specific amplification; OSCC, oral squamous cell carcinoma; HNSCC, head and neck squamous cell carcinoma; HCC, hepatic cell carcinoma; NPC, Nasopharyngeal Carcinoma; NSCC, Non small cell Lung cancer; PTC, Papillary Thyroid Cancer.

All studies were case-control studies, including 40 studies on 10 breast cancer, 7 gastric cancer, 7 colorectal cancer, 4 lung cancer, and 12 on other cancer types. There were 28 studies of Asian descendent, 11 of Caucasian descendent. A classic PCR-RFLP assay was used in 17 out of 40 studies, the other molecular genotyping methods, such as Taqman, MassARRAY, and HRM, were used in other studies. 32 studies were randomly repeated a portion of samples as quality control while genotyping.

### Quality assessment

According to the NOS for quality of case-control, the study-specific quality scores are summarized in Table 2. A star system of the NOS (range, 0–9 scores) has been developed for the evaluation, and the quality scores ranged from 4 to 8. The average scores of case-control studies were 6.49.

**Table 2:** Quality assessment of included studies based on the newcastle–ottawa scale

Study	Selection (score)				Comparability (score)	Exposure (score)			Total score^b^
	Adequate definition of patient case	Representativeness of patients cases	Selection of controls	Definition of control	Control for important factor or additional factor	Ascertainment of exposure (blinding)	Same method of ascertainment for participants	Non-response rate^a^	
He BS [11]	1	1	1	1	2	0	1	0	**7**
Du ML [12]	1	1	1	1	2	0	1	0	**7**
Dikeakos P [13]	1	1	0	1	2	0	1	1	**7**
Wei WJ [15]	1	1	1	1	2	0	1	0	**7**
Wang R [16]	1	1	0	1	1	0	1	0	**5**
Huang GL [18]	1	1	1	1	2	0	1	0	**7**
Song XC [21]	1	1	1	1	2	0	1	1	**8**
Tu HF [22]	1	1	0	1	2	0	1	0	**6**
Zhang M [23]	1	1	1	1	2	0	1	0	**7**
Zhang MW [24]	1	1	1	1	2	0	1	0	**7**
Min KT [25]	1	1	1	1	2	0	1	0	**7**
Aho DH [26]	1	1	1	1	2	0	1	1	**8**
Kim WH [27]	1	1	1	1	2	0	1	0	**7**
Vinci S [28]	1	1	1	1	1	0	1	0	**6**
Vinci S [29]	1	1	0	1	2	0	1	0	**7**
Li PY [30]	1	1	0	1	2	0	1	0	**6**
Zhang MW [31]	1	1	1	1	2	0	1	0	**7**
Liu ZS [32]	1	1	1	1	2	0	1	1	**8**
Tian T [33]	1	1	1	1	2	0	1	0	**7**
Wang ZW [34]	1	1	1	1	1	0	1	0	**6**
Qi P [36]	1	1	1	1	2	0	1	0	**7**
Yin ZH [37]	1	1	0	1	2	0	1	0	**6**
Cao Y [38]	1	1	1	1	2	0	1	0	**7**
Kupcinskas J (C) [39]	1	1	0	1	0	0	1	0	**4**
Kupcinskas J (G) [40]	1	1	0	1	2	0	1	0	**6**
Song B [41]	1	1	1	1	2	0	1	0	**7**
Zhang JJ [42]	1	1	1	1	2	0	1	0	**7**
Zhang N [43]	1	1	1	1	2	0	1	0	**7**
Catucci I [44]	1	1	0	1	1	0	1	0	**5**
Hezova R [45]	1	1	1	1	1	0	1	0	**6**
Shi DN [46]	1	1	1	1	2	0	1	0	**7**
Zhang MW [47]	1	1	1	1	0	0	1	0	**5**
Zhou Y [49]	1	1	0	1	1	0	1	0	**5**
Zhang P [50]	1	1	1	1	1	0	1	0	**6**
Sun QM [51]	1	1	0	1	2	0	1	0	**6**
Kontorovich T [52]	1	1	1	1	0	0	1	0	**5**
Yang RX [53]	1	1	1	1	2	0	1	0	**7**

a When there was no statistical significance in the response rate between case and control groups by using a chi-squared test (*P* > 0.05), one point was awarded.

b Total score was calculated by adding up the points awarded in each item.

### Quantitative data synthesis

**Figure 2:**
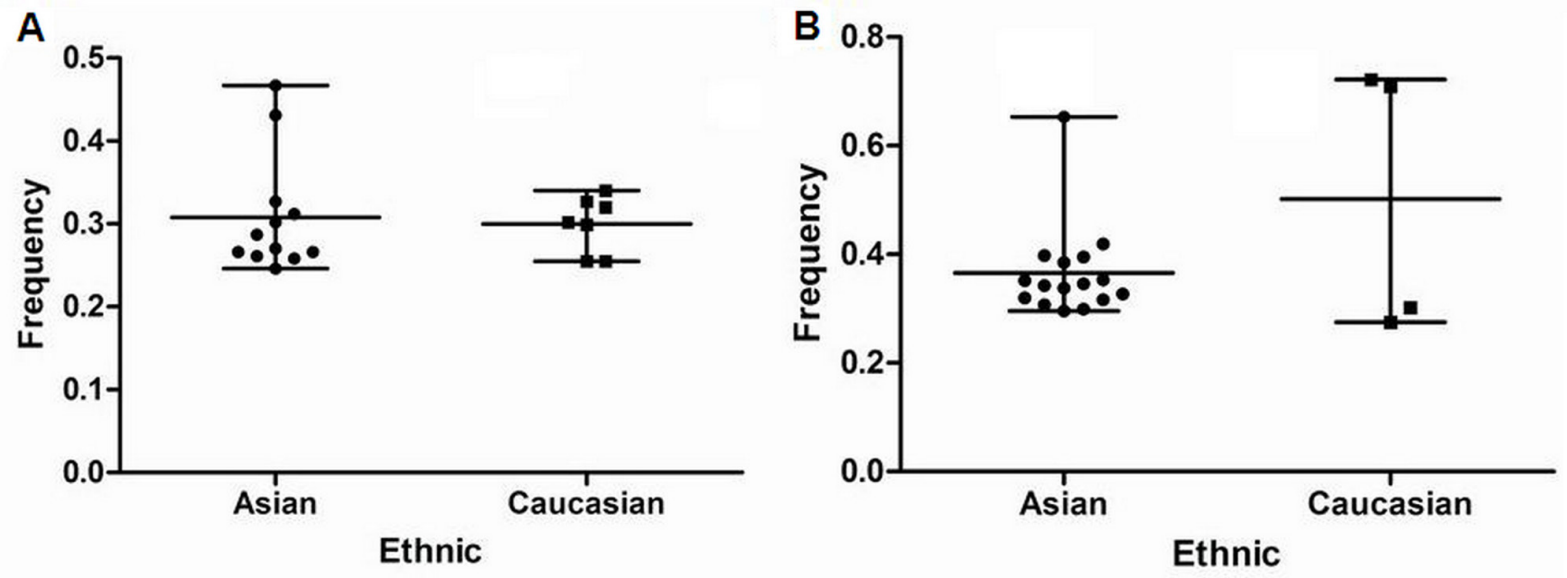
(A) frequencies of C allele in rs2292832 among controls stratified by ethnicity (B) frequencies of G allele in rs895819 among controls stratified by ethnicity

For all of control subjects included in this study, the frequencies of risk C allele in rs2292832 for Caucasians and Asians were 33.66% (Mean ± SEM, 33.66% ± 2.18%) and 50.20% (Mean ± SEM, 50.20% ± 12.34%) (Figure 2A). The frequencies of risk G allele in rs895819 for Caucasians and Asians were 30.78% (Mean ± SEM, 30.78% ± 2.04%) and 29.63% (Mean ± SEM, 29.63% ± 1.45%) (Figure 2B). The frequencies of risk C allele in rs2292832 varied greatly among different control populations (*P* = 0.00).

**Figure 3:**
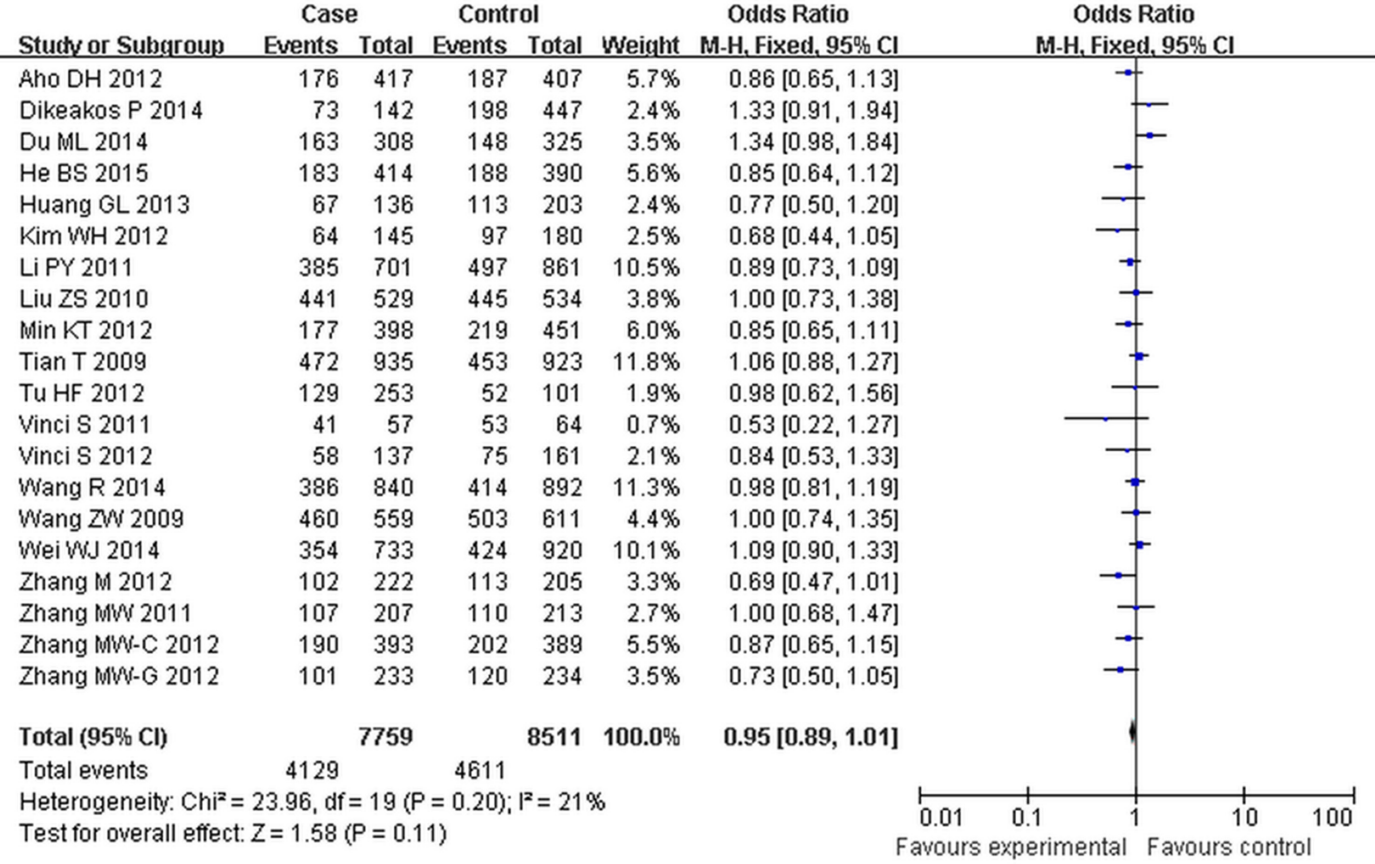
Forest plot of cancer risk associated with rs2292832 for the recessive model (CT vs TT) The squares and horizontal lines correspond to the study-specific OR and 95% CI. The area of the squares reflects the study specific weight. The diamond represents the pooled OR and 95% CI.

For the rs2292832 polymorphism, no significant risk association was observed in the overall pooled analysis (Table 3, Figure 3). When grouped by the cancer types, significant associations were found in breast cancer (CT + CC vs TT: OR = 0.83, 95% CI: 0.70–0.98, *P =* 0.03; CC vs CT + TT: OR = 0.80, 95% CI: 0.68–0.93, *P* = 0.00) (Table 4).

**Table 3:** Main results of pooled ORs of the rs2292832 and rs895819 polymorphisms on cancer risk in the meta-analysis

comparisons	Cases	Controls	Heterogeneity test			Summary OR (*95% CI*)	Hypothesis test		Studies
	n/N	n/N	*Q*	*P*	*I^2^* (%)		*Z*	*P*	
rs2292832									
C vs T	7995/19596	8591/20464	20.34	0.09	36	0.93 (0.84,1.06)	0.52	0.13	20
CT vs TT	4129/7759	4611/8511	23.96	0.20	21	0.95 (0.89,1.01)	1.58	0.11	20
CC vs TT	1910/5536	2020/5820	21.82	0.06	40	0.97 (0.82,1.14)	0.40	0.69	20
CT + CC vs TT	6039/9669	6650/10550	32.71	0.01	44	0.93 (0.85,1.01)	0.68	0.09	20
CC vs CT + TT	2068/9994	2182/10757	47.55	< 0.01	51	1.00 (0.88,1.14)	0.08	0.94	21
rs895819									
G vs A	4725/15804	5412/17610	43.16	< 0.01	58	0.99 (0.91,1.17)	0.09	0.93	19
AG vs AA	3179/7062	3692/7976	30.95	0.03	45	0.99 (0.88,1.12)	0.19	0.85	19
GG vs AA	798/4681	873/5217	27.45	0.04	42	1.07 (0.91,1.26)	0.80	0.42	19
AG + GG vs AA	3987800	4464/9060	42.79	< 0.01	77	1.13 (0.97,1.31)	1.55	0.12	19
GG vs AG + AA	798/7770	873/8911	37.20	0.01	52	1.06 (0.90,1.25)	0.69	0.49	19

**Table 4:** Stratified analyses of rs2292832 polymorphism on cancer risk

Comparisons	Heterogeneity test			Summary OR (*95% CI*)	Hypothesis test		Studies
	*Q*	*P*	*I*^2^ (*%*)		*Z*	*P*	
Ethnic Asian							
C vs T	51.04	< 0.01	49	0.90 (0.81,1.01)	1.86	0.06	16
CT vs TT	18.78	0.22	20	0.94 (0.88,1.01)	1.70	0.09	16
CC vs TT	33.84	0.01	41	0.93 (0.78,1.11)	0.79	0.43	16
CT + CC vs TT	3.93	0.02	44	0.94 (0.87,1.03)	1.31	0.19	16
CC vs CT + TT	32.41	0.02	38	1.00 (0.88,1.14)	0.08	0.94	16
Caucasian							
C vs T	2.55	0.28	22	1.06 (0.84,1.33)	0.47	0.63	4
CT vs TT	4.73	0.19	37	1.02 (0.82,1.25)	0.14	0.89	4
CC vs TT	10.45	0.02	61	1.16 (0.67,2.01)	0.54	0.59	4
CT + CC vs TT	6.09	0.11	11	1.08 (0.88,1.31)	0.72	0.47	4
CC vs CT + TT	8.12	0.09	51	1.10 (0.86,1.41)	0.79	0.43	5
Cancer types							
Colorectal Cancer							
C vs T	0.79	0.67	0	0.97 (0.85,1.10)	0.48	0.63	3
CT vs TT	0.02	0.99	0	0.85 (0.71,1.02)	1.72	0.09	3
CC vs TT	1.02	0.60	0	0.94 (0.71,1.25)	0.42	0.68	3
CT + CC vs TT	1.12	0.57	0	0.87 (0.67,1.15)	0.97	0.33	3
CC vs CT + TT	0.32	0.96	0	1.13 (0.97,1.33)	1.56	0.12	3
Lung Cancer							
C vs T	3.65	0.16	45	0.97 (0.86,1.08)	0.63	0.53	3
CT vs TT	1.99	0.37	0	0.86 (0.67,1.11)	1.14	0.25	3
CC vs TT	4.43	0.11	55	0.93 (0.73,1.20)	0.53	0.60	3
CT + CC vs TT	1.62	0.44	0	1.03 (0.83,1.28)	0.25	0.80	3
CC vs CT + TT	3.28	0.19	39	0.96 (0.83,1.12)	0.48	0.63	3
Breast Cancer							
C vs T	13.72	< 0.01	55	0.82 (0.61,1.10)	1.31	0.19	3
CT vs TT	2.19	0.33	9	0.86 (0.72,1.03)	1.64	0.10	3
CC vs TT	5.81	0.55	46	0.82 (0.65,1.03)	1.73	0.08	3
CT + CC vs TT	2.72	0.26	26	0.83 (0.70,0.98)	2.18	0.03	3
CC vs CT + TT	2.82	0.24	29	0.80 (0.68,0.93)	2.81	0.00	3
Other cancers							
C vs T	13.42	0.06	45	0.91 (0.78,1.05)	1.29	0.20	11
CT vs TT	19.35	0.04	48	0.96 (0.85,1.08)	0.75	0.45	11
CC vs TT	16.28	0.02	57	1.06 (0.83,1.35)	0.47	0.64	11
CT + CC vs TT	13.67	0.09	41	1.06 (0.96,1.16)	1.17	0.24	11
CC vs CT + TT	5.98	0.54	0	1.18 (1.06,1.31)	3.14	0.00	12
Source of control Population							
C vs T	78.91	< 0.01	60	0.92 (0.83,1.02)	1.53	0.13	17
CT vs TT	20.50	0.20	22	0.95 (0.88,1.01)	1.59	0.11	17
CC vs TT	29.47	0.02	46	1.00 (0.86,1.16)	0.04	0.97	17
CT + CC vs TT	26.00	0.05	38	0.96 (0.90,1.03)	1.06	0.29	17
CC vs CT + TT	27.06	0.06	38	1.01 (0.94,1.10)	0.32	0.75	18
Hospital							
C vs T	13.71	0.01	65	0.97 (0.68,1.38)	0.17	0.86	3
CT vs TT	3.34	0.19	40	0.98 (0.83,1.15)	0.30	0.77	3
CC vs TT	17.29	< 0.01	68	0.83 (0.64,2.03)	0.40	0.69	3
CT + CC vs TT	7.75	0.02	64	0.99 (0.69,1.43)	0.05	0.96	3
CC vs CT + TT	15.24	< 0.01	67	0.82 (0.57,1.80)	0.49	0.62	3
Sample size							
≥ 300							
C vs T	76.76	< 0.01	66	0.99 (0.87,1.12)	0.19	0.85	12
CT vs TT	12.83	0.30	14	0.99 (0.92,1.06)	0.34	0.74	12
CC vs TT	35.37	< 0.01	59	1.04 (0.86,1.26)	0.42	0.68	12
CT + CC vs TT	21.90	0.03	50	1.00 (0.91,1.10)	0.04	0.97	12
CC vs CT + TT	30.33	< 0.01	64	1.03 (0.90,1.19)	0.47	0.64	13
< 300							
C vs T	7.50	0.38	7	0.92 (0.94,1.11)	1.88	0.06	8
CT vs TT	4.34	0.74	0	0.89 (0.78,1.02)	1.74	0.08	8
CC vs TT	12.99	0.07	46	0.82 (0.65,1.04)	1.66	0.10	8
CT + CC vs TT	5.03	0.66	0	0.90 (0.80,1.03)	1.70	0.09	8
CC vs CT + TT	13.13	0.07	47	0.93 (0.75,1.14)	0.73	0.47	8

**Figure 4:**
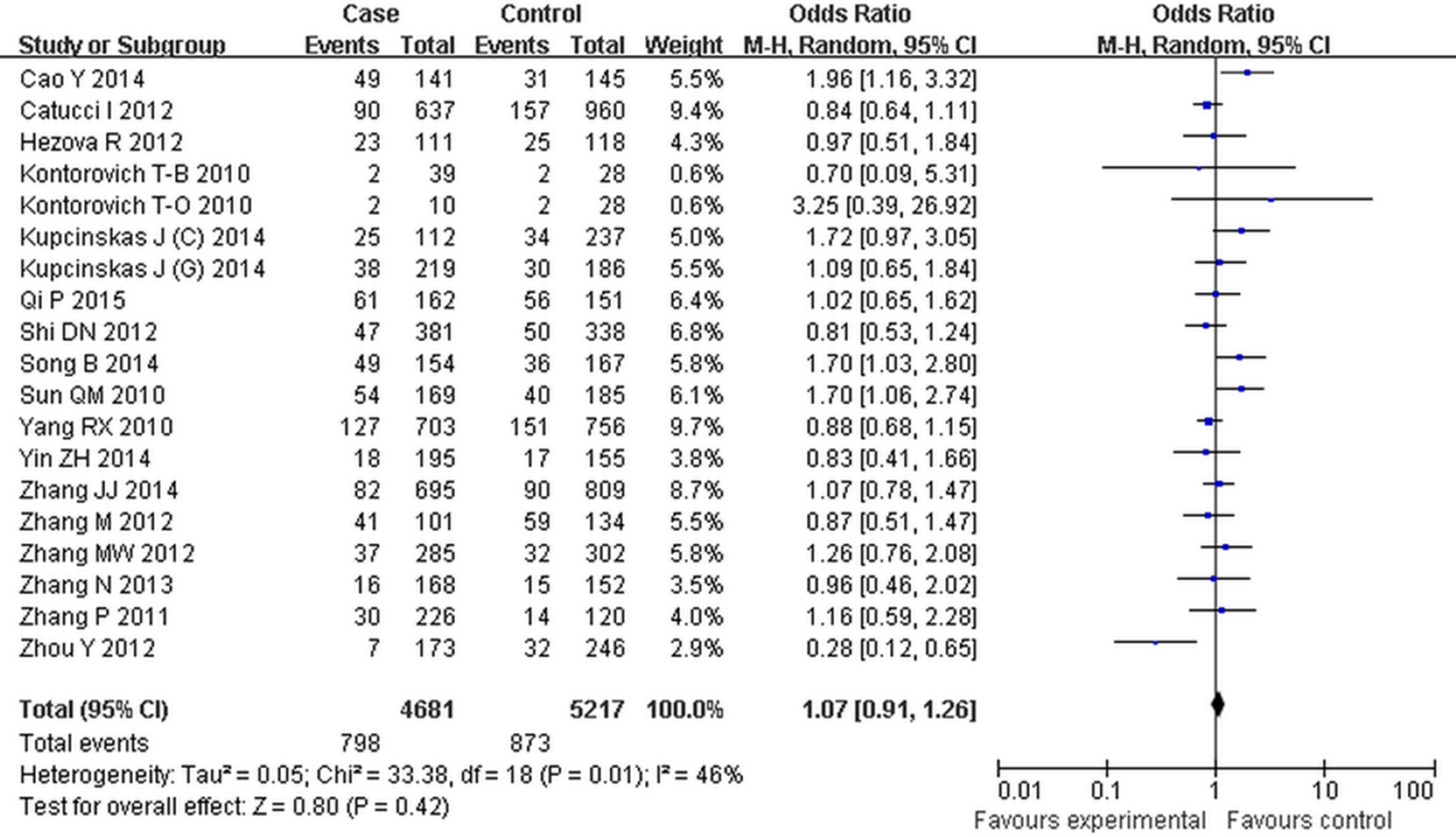
Forest plot of cancer risk associated with rs895819 for the GG vs AA compared with the AA genotype

For the rs895819 polymorphism, we failed to find any associations between rs895819 polymorphism and cancer risk (Table 3, Figure 4). In the subgroup analysis by ethnicity, statistically significantly reduced cancer risks were found among Asian for dominant contrast (AG + GG vs AA: OR = 1.24, 95% CI: 1.03–1.50, *P* = 0.02) (Table 5). In contrast, a subtly decreased risk was observed in the Caucasian population (G vs A: OR = 0.92, 95% CI: 0.85–0.99, *P* = 0.03; AG vs AA: OR = 0.92, 95% CI: 0.85–0.99, *P* = 0.00) (Table 5). Subgroup analysis by cancer types revealed a decreased risk in breast cancer (G vs A: OR = 0.92, 95% CI: 0.86–0.99, *P* = 0.03; AG vs AA: OR = 0.83, 95% CI: 0.75–0.92, *P* < 0.01; AG + GG vs AA: OR = 0.88, 95% CI: 0.80–0.97, *P* = 0.01), whereas a significantly increased risk was observed in colorectal cancer (GG vs AA: OR = 1.45, 95% CI: 1.10–1.92, *P* < 0.01; AG + GG vs AA: OR = 1.35, 95% CI: 1.15–1.58, *P* < 0.01; GG vs AG + AA: OR = 1.36, 95% CI: 1.04–1.77, *P* = 0.02) (Table 5).

**Table 5:** Stratified analyses of the rs895819 polymorphism on cancer risk

Comparisons	Heterogeneity test			Summary OR (*95% CI*)	Hypothesis test		Studies
	*Q*	*P*	*I^2^* (*%*)		*Z*	*P*	
Ethnic							
Asian							
G vs A	34.11	< 0.01	68	1.02 (0.91,1.14)	0.27	0.79	12
AG vs AA	27.19	0.01	60	1.09 (0.95,1.26)	1.25	0.21	12
GG vs AA	24.68	0.01	55	1.09 (0.87,1.37)	0.73	0.47	12
AG + GG vs AA	53.69	< 0.01	80	1.24 (1.03,1.50)	2.28	0.02	12
GG vs AG + AA	30.73	< 0.01	64	1.03 (0.81,1.31)	0.25	0.80	12
Caucasian							
G vs A	6.91	0.33	13	0.92 (0.86,0.99)	2.27	0.02	7
AG vs AA	7.70	0.26	22	0.81 (0.73,0.89)	3.82	0.00	7
GG vs AA	6.74	0.35	11	0.95 (0.80,1.12)	0.65	0.51	7
AG + GG vs AA	4.17	0.65	0	0.87 (0.79,0.95)	2.69	0.00	7
GG vs AG + AA	6.47	0.37	7	1.03 (0.88,1.02)	0.34	0.74	7
Breast cancer							
G vs A	8.76	0.12	43	0.92 (0.86,0.99)	2.15	0.03	6
AG vs AA	11.41	0.04	56	0.83 (0.75,0.92)	3.51	0.00	6
GG vs AA	1.17	0.95	0	0.90 (0.76,1.07)	1.21	0.23	6
AG + GG vs AA	5.80	0.33	14	0.88 (0.80,0.97)	2.58	0.01	6
GG vs AG + AA	2.40	0.79	0	0.98 (0.84,1.15)	0.24	0.81	6
Gastric cancer							
G vs A	16.96	0.00	62	1.11 (0.84,1.46)	0.70	0.48	4
AG vs AA	10.15	0.02	50	1.08 (0.80,1.47)	0.50	0.42	4
GG vs AA	15.44	0.00	60	1.05 (0.55,1.99)	0.15	0.88	4
AG + GG vs AA	13.52	0.00	58	1.10 (0.79,1.53)	0.55	0.58	4
GG vs AG + AA	12.52	0.01	56	1.02 (0.59,1.76)	0.07	0.94	4
Colorectal Cancer							
G vs A	1.78	0.62	0	1.07 (0.94,1.21)	1.06	0.29	4
AG vs AA	3.42	0.33	12	1.14 (0.96,1.35)	1.47	0.14	4
GG vs AA	3.40	0.33	12	1.45 (1.10,1.92)	2.66	0.00	4
AG + GG vs AA	7.81	0.05	62	1.35 (1.15,1.58)	3.65	0.00	4
GG vs AG + AA	2.52	0.47	0	1.36 (1.04,1.77)	2.27	0.02	4
Other cancers							
G vs A	2.12	0.55	0	0.87 (0.79,0.96)	2.87	0.00	4
AG vs AA	7.08	0.07	58	0.92 (0.81,1.04)	1.30	0.19	4
GG vs AA	2.49	0.48	0	0.96 (0.76,1.22)	0.30	0.77	4
AG + GG vs AA	22.87	0.00	70	1.26 (0.77,2.07)	0.92	0.36	4
GG vs AG + AA	1.70	0.64	0	1.05 (0.84,1.33)	0.45	0.65	4
Source of control Population							
G vs A	28.89	0.01	58	0.99 (0.90,1.10)	0.18	0.86	13
AG vs AA	43.20	0.00	72	1.02 (0.86,1.21)	0.22	0.83	13
GG vs AA	14.44	0.27	17	1.06 (0.93,1.21)	0.83	0.41	13
AG + GG vs AA	61.57	0.00	81	1.14 (0.94,1.38)	1.36	0.17	13
GG vs AG + AA	20.53	0.06	42	1.03 (0.91,1.17)	0.46	0.65	13
Hospital							
G vs A	14.18	0.01	65	0.99 (0.86,1.15)	0.08	0.94	6
AG vs AA	7.78	0.17	36	0.94 (0.84,1. 05)	1.11	0.27	6
GG vs AA	18.75	0.00	73	0.98 (0.65,1.49)	0.08	0.94	6
AG + GG vs AA	27.21	0.00	82	1.10 (0.84,1.43)	0.68	0.50	6
GG vs AG + AA	16.68	0.01	70	1.06 (0.73,1.55)	0.32	0.75	6
Sample size							
≥ 300							
G vs A	22.21	0.02	59	0.95 (0.87,1.04)	1.16	0.25	10
AG vs AA	27.95	0.01	68	0.92 (0.80,1.05)	1.23	0.22	10
GG vs AA	21.34	0.01	58	0.99 (0.80,1.23)	0.05	0.96	10
AG + GG vs AA	76.99	0.00	88	1.09 (0.88,1.35)	0.77	0.44	10
GG vs AG + AA	17.22	0.05	48	1.03 (0.91,1.16)	0.42	0.67	10
< 300							
G vs A	13.95	0.08	43	1.08 (0.98,1.18)	1.45	0.15	9
AG vs AA	12.81	0.12	38	1.15 (1.00,1.33)	2.02	0.04	9
GG vs AA	8.96	0.35	11	1.22 (0.99,1.50)	1.85	0.06	9
AG + GG vs AA	9.82	0.28	19	1.19 (0.98,1.32)	1.74	0.07	9
GG vs AG + AA	19.99	0.01	60	1.08 (0.77,1.50)	0.44	0.66	9

### Test of heterogeneity

In the overall pooled analysis, the results showed that both rs2292832 and rs895819 had heterogeneity in part of genotype with *P* value less than 0.05. Therefore, we analyzed the summary ORs with random-effect models if the heterogeneity existed. Fixed-effect models were used to analyze the summary odds ratios for the rest. Subsequently, meta regression in Stata12.0 was used to assess the source of heterogeneity for rs2292832 and rs895819, including publication year, ethnicity (Asians, Caucasians), cancer type, matched controls (yes or not), language (English or Chinese), source of control (hospital or population), assay, sample size (300 as the boundary) and quality control (with or without). It was detected that the systemic results were not altered by these characteristics (Table 6).

**Table 6:** The results of heterogeneity test for rs2292832 and rs895819

Comparisons	Publication year	Ethnicity	Cancer type	Match	Language	Source of control	Assay	Sample size	Quality control
rs2292832									
C vs T	0.737	0.339	0.256	0.812	0.653	0.547	0.417	0.291	0.781
CT vs TT	0.392	0.440	0.331	0.329	0.220	0.514	0.519	0.765	0.529
CC vs TT	0.388	0.838	0.463	0.784	0.463	0.875	0.772	0.573	0.514
CT + CC vs TT	0.737	0.440	0.547	0.956	0.853	0.443	0.949	0.552	0.554
CC vs CT + TT	0.519	0.519	0.440	0.331	0.389	0.396	0.838	0.336	0.815
rs895819									
G vs A	0.418	0.426	0.275	0.581	0.593	0.581	0.336	0.581	0.225
AG vs AA	0.440	0.841	0.415	0.797	0.596	0.797	0.554	0.797	0.442
GG vs AA	0.838	0.721	0.487	0.998	0.827	0.498	0.423	0.998	0.366
AG + GG vs AA	0.418	0.426	0.159	0.989	0.656	0.989	0.359	0.989	0.396
GG vs AG + AA	0.327	0.841	0.881	0.077	0.914	0.077	0.073	0.077	0.990

### Evaluation of publication bias

**Figure 5:**
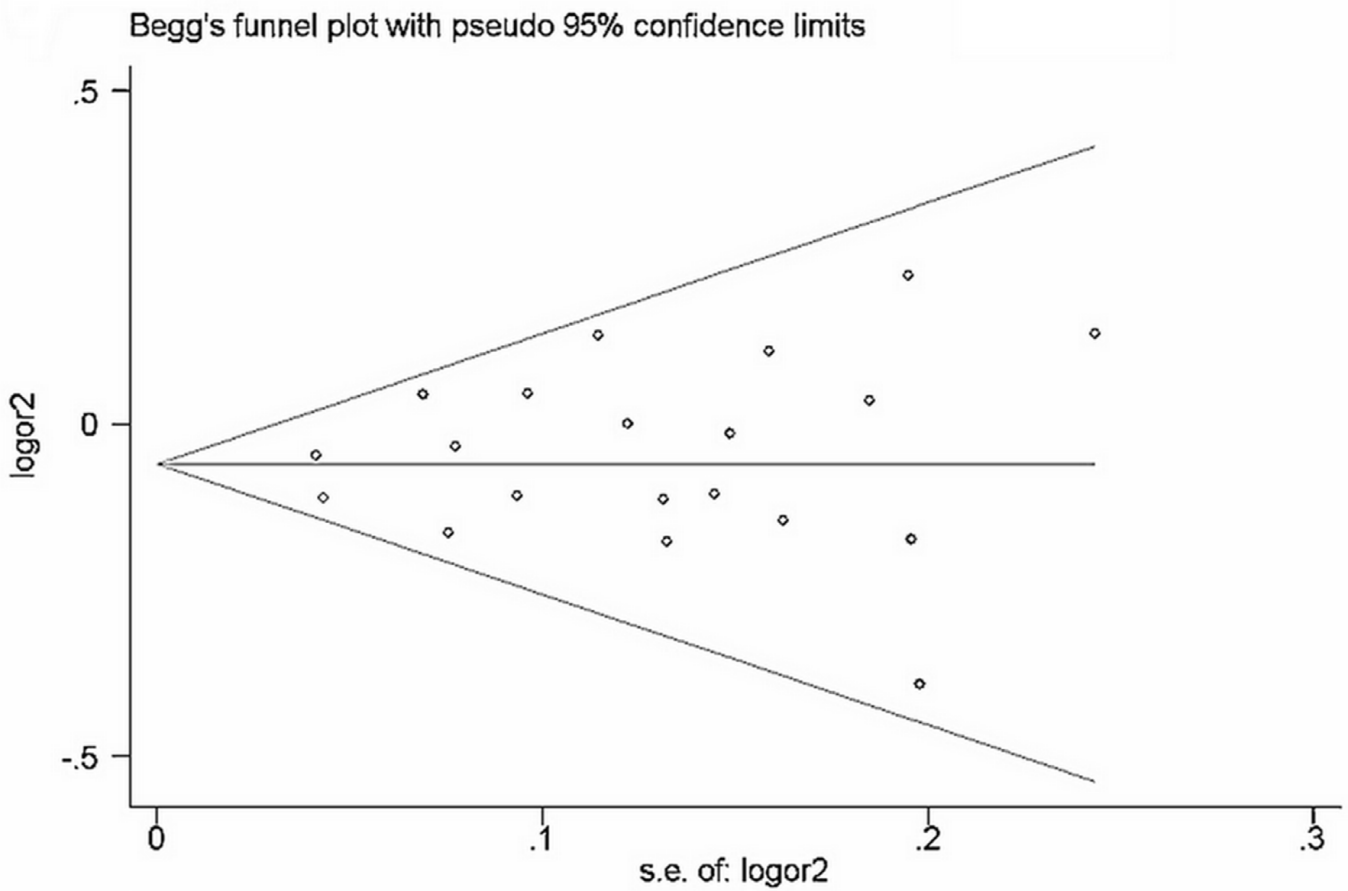
Funnel plot of rs2292832 polymorphism and cancer risk for dominant models (TT + CT vs CC) The horizontal line in the funnel plot indicates the fixed-effects summary estimate, whereas the sloping lines indicate the expected 95% CI for a given SE.

**Figure 6:**
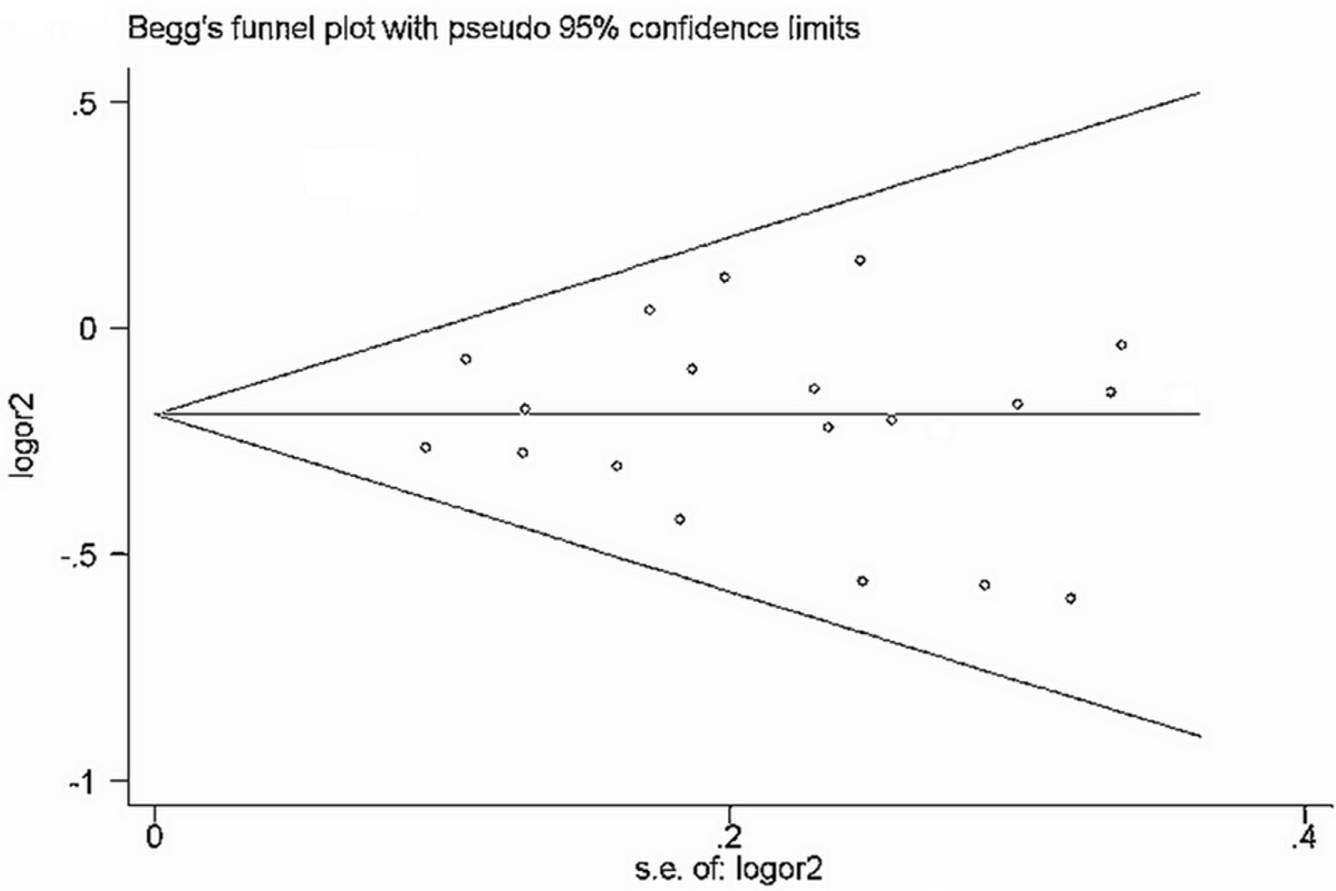
Funnel plot of rs895819 polymorphism and cancer risk for dominant models (TT + CT vs CC)

Begg's funnel plot and Egger's test (Table 7) were performed to assess the publication bias of the currently available literature. The shape of the funnel plots did not reveal any evidence of obvious asymmetry in all comparison models (Figure 5 and Figure 6).

**Table 7:** Publication bias of rs2292832 and rs895819 for Egger's test

Comparisons	*t*	*p*	*95% CI*
rs2292832			
T vs C	0.96	0.358	−1.657∼4.245
CT vs CC	−0.45	0.661	−1.748∼1.151
TT vs CC	0.96	0.358	−1.171∼3.001
CT + TT vs CC	0.37	0.715	−1.256∼1.777
TT vs CT + CC	1.60	0.083	−0.572∼3.100
rs895819			
G vs A	0.44	0.673	−2.337∼3.452
AG vs AA	1.18	0.270	−1.122∼3.555
GG vs AA	0.28	0.789	−1.792∼2.291
AG + GG vs AA	1.12	0.292	−1.219∼3.612
GG vs AG + AA	−0.07	0.943	−1.923∼1.803

### Sensitivity analysis

A single study included in the meta-analysis was deleted each time to reflect the influence of the individual data set to the pooled ORs, and the corresponding pooled ORs were not materially changed (data not shown).

## DISCUSSION

In the present study, an association between the two common SNPs in microRNAs (rs2292832 and rs895819) and cancer risk was evaluated by the pooled results from 40 published studies. The results demonstrated that the rs2292832 was associated with a significantly reduced risk for developing cancer in the breast cancer (dominant and recessive model), and for the rs895819 G allele, AG genotype and dominant model were associated with a decreased risk for Caucasian population and breast cancer, in contrast, a subtly increased risk was observed in a Asian population (dominant model) and colorectal cancer (GG genotype, dominant model and recessive model).

Thus far, for the rs2292832, no significant association was observed in overall pooled results [54, 55]. In contrast to the published results, this study revealed the different association between rs2292832 polymorphism and breast cancer risk. This suggests that the molecular mechanisms underlying the genetic associations of miRNA-SNPs with cancer are complex and vary by cancer site. Considering the influence of the T allele in rs2292832 might be masked by the presence of other as-yet unidentified causal genes involved in cancer development on this polymorphism [56], our results should be interpreted with caution, and more studies will need to be analyzed to confirm the results.

The rs895819 is well recognized to be involved in the pathogenesis, metastasis, and invasion of multiple cancer types, by functioning as an oncogene via complex mechanisms [57–59]. The rs895819, as an oncomiR, exhibited its oncogenic activity through regulating target genes [60, 61]. It means that down-regulation of miR-27a may contribute to decreased cancer risk through up-regulating the targets. Although the binding of the mature miRNA to target mRNAs was not influenced by the rs895819 [62], some published studies had demonstrated that polymorphisms in premiRNAs could influence the expression of their mature forms, as well as were involved in the binding of some nuclear factors in miRNA processing [63]. Therefore, we presumed that rs895819 affected the processing or/and expression of miR-27a, which resulted in down-regulation of miR-27a. The presumption was supported by our findings in breast cancer subgroup.

This comprehensive and updated meta-analysis further support the rs895819 G allele was associated with a decreased risk for breast cancer, whereas a subtly increased risk was observed in colorectal cancer. In addition, significant associations with an increased risk for the Caucasian population, but a significantly reduced risk for the Asian population, suggesting a possible ethnic difference in the genetic background and the environment, which was the similar to that reported by Wang et al. [64] and Zhong et al. [65]. However, the risk of different cancer types and multiethnic should be confirmed by more studies.

Although meta-analysis is robust, our study still has some limitations. Firstly, we pooled the data based on unadjusted information and lack the consideration of combination genetic factors together with environmental exposures, while a more precise analysis needs to be conducted if individual data are available. Secondly, although all eligible studies were summarized, the relatively small sample size of studies may lead to reduced statistical power when stratified according to the cancer type or ethnicity. Thirdly, the different genotyping strategies may contribute to the bias in the analysis. Fourthly, Publication bias may exist, because only published studies were included in this meta-analysis, although the result for publication bias was not statistically significant. Finally, the data sets without excluding the studies with inefficient scores base on NOS.

In summary, current data suggest that the rs2292832 polymorphism may contribute to increased susceptibility to breast cancer, and the rs895819 polymorphism was a protective factor for cancer development among Caucasian and may contribute to breast and colorectal cancer susceptibility. Further multi-centric studies are still needed to confirm the present results.

## MATERIALS AND METHODS

### Identification of eligible studies

A comprehensive literature search was conducted using the PubMed, Springer, Elsevier, CNKI (Chinese), and Wanfang (Chinese) Digital Dissertations Databases for relevant articles published in English or Chinese up to July 2015 with key words ‘microRNA/miR-149/miR-27a’, ‘rs2292832/rs895819’,‘polymorphism’, and ‘cancer’. The full text of the candidate articles were examined carefully to determine whether they accorded with the inclusion criteria for the meta-analysis. The present study was conducted in accordance with PRISMA guidelines [66].

The inclusion criteria were as follows: 1) about the rs2292832/rs895819 polymorphisms and cancer risk, 2) based on case-control studies (including cohort studies), 3) sufficient published data for estimating an odds ratio (OR) with 95% confidence interval (CI), and 4) genotype distribution of control groups must be in accordance with the assumptions of Hardy-Weinberg equilibrium (HWE).

In case of redundant publications, only the studies with the largest sample size and/or latest published date were included.

### Data extraction

Data were extracted independently by two investigators (YJF and FJD). Data for analyses, including first author, publication year, cancer type, country of origin, ethnicity, study design, genotype detection methods and quality control or not. If discrepancies existed, consensus would be finally reached on discussion.

### Quality assessment

Quality assessment criteria were utilized to evaluate methodological quality of included studies based on Newcastle-Ottawa Scale (NOS) [67] for quality of case-control. A nine-point scale of the NOS (range, 0–9 points) has been developed for the evaluation, a high-quality study was defined as one with a score of ≥ 7.

### Statistical analysis

The analyses were conducted in Review Manager 5.0 (Version 5 for Windows, Cochrane Collaboration, Oxford, UK). The overall strength of an association between rs2292832 and rs895819 polymorphisms and cancer risk assessed by crude ORs together with their corresponding 95% CIs. The stratified analysis was conducted by ethnicity (Asians, Caucasians), cancer type, source of control and sample size (300 as the boundary).

Heterogeneity in meta-analysis refers to the variation in study outcomes between different studies. Between-study heterogeneity was evaluated with a χ^*2*^ based *Q*-test among the studies [68]. Heterogeneity was considered significant when *P < 0.05*. In case of no significant heterogeneity, point estimates and 95% CI was estimated using the fixed effect model (Mantel-Haenszel), otherwise, random effects model (DerSimonian Laird) was employed [69, 70]. The significance of overall OR was determined by the *Z*-test.

If there were significant heterogeneity among included studies, the sources of heterogeneity would be explored using meta-regression in Stata 12.0 (StataCorp, College Station, TX, USA). To assess the stability of the results, one-way sensitivity analyses were performed to assess the stability of the results, in which a single study in the meta-analysis was deleted each time to reflect the influence of the individual data set to the pooled OR. The publication bias was diagnosed by using inverted funnel plots, Begg's test and the Egger's test by Stata 12.0. Statistical tests performed in the present analysis were considered significant whenever the corresponding null-hypothesis probability was *P* < 0.05.
